# From triplex to tetraplex: evaluation of the diagnostic accuracy of the new Roche Cobas Liat SARS-CoV-2, influenza A/B & RSV assay

**DOI:** 10.1128/jcm.00274-26

**Published:** 2026-07-20

**Authors:** Bruno Luukinen, Mika Lång, Maria Peltola, Arttu Soinila, Tiina Jarvala, Tapio Seiskari, Minna Paloniemi

**Affiliations:** 1 Fimlab Laboratoriot 277009 , Tampere, Finland; 2 Faculty of Medicine, University of Helsinki 3835, Helsinki, Finland; 3 Faculty of Agriculture and Forestry, University of Helsinki 3835, Helsinki, Finland; 4 Faculty of Medicine and Health Technology, Tampere University 101287, Tampere, Finland; Vanderbilt University Medical Center, Nashville, Tennessee, USA

**Keywords:** molecular diagnostics, point-of-care, SARS-CoV-2, respiratory syncytial virus, influenza, respiratory infections

## Abstract

Rapid and accurate diagnostics of epidemic respiratory viruses are relevant for optimal care pathways. In this observational diagnostic accuracy study, the novel Cobas Liat assay (Roche Molecular Systems) was evaluated for the detection of SARS-CoV-2, influenza A and B, and respiratory syncytial virus (RSV) in the clinical setting of Tampere University Hospital, Tampere, Finland. In addition, the added value of the tetraplex assay instead of two partially overlapping triplex assays (influenza A/B and SARS-CoV-2 or RSV) was assessed in the context of adult and pediatric emergency department (AED, PED) point-of-care (PoC) testing environments. A total of 315 clinical nasopharyngeal swab samples were analyzed. Diagnostic accuracy was evaluated against a molecular diagnostic reference. The evaluated Cobas Liat assay had sensitivities and specificities of 100% and 98.5% for influenza A, 100% and 99.7% for influenza B, 100% and 98.2% for SARS-CoV-2, and 100% and 97.0% for RSV, respectively. Among the index and reference tests, the Cobas Liat assay demonstrated the highest analytical sensitivity for influenza A, SARS-CoV-2, and RSV. No cross-reactivity with off-panel microbes was observed. With respect to clinical applicability, implementation of the evaluated tetraplex test would decrease double testing with two triplex tests in PED, likely decreasing costs and hands-on work. In AED, tetraplex testing would likely significantly increase the diagnostic yield for RSV, only rarely covered by PoC testing in AED. The evaluated Cobas Liat assay had excellent clinical and analytical accuracy, being highly suitable for PoC use but also for laboratory-based testing of influenza viruses, SARS-CoV-2, and RSV.

**IMPORTANCE:**

Until recently, rapid point-of-care diagnostics have been somewhat limited by triplex four-channel analysis technology, separating four of the most important epidemic respiratory viruses—influenza A, influenza B, respiratory syncytial virus, and SARS-CoV-2—into two individual triplex tests. The evaluated tetraplex respiratory viral assay combines the two partially overlapping tests into a single test, streamlining analysis in two-test settings and potentially reducing diagnostic gaps in one-test settings. To our knowledge, this is the first study to describe the diagnostic accuracy of the novel Cobas Liat SARS-CoV-2, influenza A/B, and respiratory syncytial virus (RSV) assay with additional considerations for analytical sensitivity and specificity, and clinical utility in adult and pediatric emergency department use.

## INTRODUCTION

Respiratory viruses circulate globally, affecting all age groups with an emphasized burden on young children and the elderly (1, 2). Among these, influenza viruses and coronaviruses have the potential to evolve into pandemic strains, with the most recent examples being SARS-CoV-2 in 2019 and the swine flu (H1N1pdm) in 2009 (3, 4). Although SARS-CoV-2 is no longer regarded as a pandemic, it continues to have a significant clinical impact and relevance (5). Respiratory syncytial virus (RSV) infections pose a significant health risk, especially for young children under the age of 5, but the elderly are also disproportionately affected (6, 7). Effective treatment, disease surveillance, and control, as well as patient management, rely on distinguishing the causative agent of an infection. Rapid turnaround times enable faster initiation of proper treatment, reduce unnecessary patient isolation, and are associated with a shorter length of stay at the emergency department (ED) (8–10). An accurate and timely diagnosis prevents and minimizes the use of unnecessary antibiotics, but also guides the selection of appropriate antivirals (11). Nucleic acid amplification tests are widely adopted by clinical microbiology laboratories because they offer unmatched performance when diagnosing viral respiratory infections (12).

In this study, we evaluated the diagnostic accuracy of the Cobas Liat SARS-CoV-2, influenza A/B, and RSV assay. As a secondary endpoint, we also evaluated the clinical utility of the tetraplex assay’s panel coverage in the primary study site, the adult and pediatric emergency departments of Tampere University Hospital, Tampere, Finland. At the time of the study, the respiratory point-of-care (PoC) diagnostics of these departments involved the use of predecessor triplex assays for Cobas Liat with partially overlapping assay panels (influenza A/B and RSV and influenza A/B and SARS-CoV-2). The objective of this study was thus to examine whether the new tetraplex Cobas Liat SARS-CoV-2, influenza A/B, and RSV assay could replace the predecessor triplex assays by streamlining the diagnostic workflow without decreasing diagnostic accuracy.

## MATERIALS AND METHODS

### Study design

This study was an observational multi-component study involving a primary cross-sectional diagnostic accuracy sub-study and a secondary descriptive registry-based sub-study (described separately).

In the diagnostic accuracy study, pre-analyzed nasopharyngeal swab (NPS) samples were used to evaluate the diagnostic accuracy of the Cobas Liat SARS-CoV-2, influenza A/B, and RSV assay (Roche Molecular Systems, Pleasanton, CA, the United States). The evaluation was performed in Fimlab Laboratoriot Clinical Microbiology Department, Tampere, Finland, with molecular respiratory infection diagnostic leftover samples originating from Wellbeing Services County of Pirkanmaa (Pirha). In the context of this study, Fimlab Laboratoriot provides clinical laboratory services to all healthcare levels primarily, but not exclusively, in public healthcare. Fimlab Laboratoriot also supervises local microbiological PoC testing. Clinical PoC samples were collected between March and May 2025 at the end of the respiratory epidemic season 2024/2025. To increase the number of samples in the diagnostic accuracy sub-study, PoC samples were supplemented with positive and negative laboratory-based clinical samples analyzed during (January to March 2025) and after the epidemic season (May to July 2025), respectively. Evaluation analyses were performed between April and July 2025. The study has been reported according to the STARD 2015 guidelines.

### Participants

Participants were originally selected from Tampere University Hospital Emergency Department (here, Adult Emergency Department, AED) and Pediatric Emergency Department (PED), Tampere, Finland, as these units perform PoC analysis using the CE IVD certified Cobas Liat respiratory triplex assays: Cobas SARS-CoV-2 and influenza A/B (SARS-CoV-2-triplex assay) and Cobas influenza A/B and RSV assays (RSV-triplex assay) (Roche Molecular Systems), both analyzed with the Cobas Liat system (Roche Molecular Systems). Age, biological sex, department (AED/PED), and analyzed tests (SCOV2- and/or RSV-triplex test) of participants were collected to describe patient demographics of the study.

Eligibility criteria for Cobas Liat respiratory triplex analysis (both EDs) were symptoms of respiratory infection and admission to a hospital ward. The AED primarily used the SARS-CoV-2-triplex assay, while the PED primarily used both RSV- and SARS-CoV-2-triplex assays. The inclusion criteria for this study included testing with at least one of the two Cobas Liat respiratory triplex assays, and NPS sampling into a 3 mL Universal Transport Media (UTM) tube (Copan Diagnostics, Murrieta, CA, the United States), which served as the test material for the evaluation study. Samples from eligible participants were cohorted according to the Cobas Liat respiratory triplex assay results (SARS-CoV-2, influenza A, influenza B, RSV, negative). Due to limited analytic capacity, only a fraction of daily PoC samples were sent from EDs to the laboratory for evaluation.

The laboratory-based samples supplemental to PoC testing samples were NPS samples in UTM, with the original clinical request for analysis using the central laboratory analysis method (described below) was included. These samples were taken at any healthcare level (primary to tertiary) in Pirha, Finland.

The actual study material involved a convenience sample selected from eligible participants. The distribution of the convenience sample reflected the ordinal incidence rates of available samples. Within cohorts, samples were randomly selected for analysis.

### Test methods

The Cobas Liat SARS-CoV-2, influenza A/B, and RSV assay, used with the Cobas Liat system, was the index test of the study. BD Respiratory Viral Panel (Becton, Dickinson and Company [BD], Franklin Lakes, NJ, the United States), used with the BD MAX system (BD), was the primary reference test of the study. Samples with discrepant results between the index and primary reference tests were subsequently analyzed with a secondary reference test, the Xpert Xpress CoV-2/Flu/RSV plus assay (Cepheid, Sunnyvale, IL, the United States), used with the GeneXpert IV system (Cepheid). In these cases, the reference result was resolved as concordance between two different tests (index test, primary reference test, secondary reference test). Both reference assays were in central laboratory production use at the time of the study. All assays were used according to the manufacturers’ instructions. Result analysis was fully based on the described *in vitro* diagnostic assays; no other clinical data were examined in the study. PoC samples were stored refrigerated for up to 12 h at EDs before being sent to the laboratory, where they were stored at −75°C. Supplemental laboratory-based samples were either stored frozen at −75°C (positive samples) or refrigerated (negative samples) after original analysis. All analyses with the index test and appropriate reference tests were performed within 24 h. Samples that were stored frozen were thawed once and analyzed without intermediate freezing and thawing between index and reference test analyses.

Relative analytical sensitivity and analytical specificity were evaluated with various test samples. For analytical sensitivity, a fivefold dilution series of Exact Diagnostics SARS-CoV-2, Flu, RSV Positive Run Control (Exact Diagnostics, Fort Worth, TX, the United States) was analyzed. For analytical specificity, three types of samples were tested: external quality assessment samples, ZeptoMetrix NATMEC-BIO control (ZeptoMetrix, Wilmington, DE, the United States), and clinical bacterial and fungal isolates identified with Vitek Prime (bioMérieux, Marcy L’Étoile, France). For culture isolates, 1:100 dilutions of a McFarland 0.5 suspension in 0.9% NaCl were used for analysis.

### Registry data collection

In the second part of the study, demographic and epidemiological registry data of respiratory molecular diagnostics in Tampere University Hospital between July 2024 and June 2025 were collected to describe the clinical context of the study. The registry data were collected using the internal data registry of Fimlab Laboratoriot.

Three different registry data samples were collected with the following eligibility criteria: (i) those analyzed with a respiratory PoC assay (SARS-CoV-2-triplex, RSV-triplex, or both) in Tampere University Hospital AED or PED, (ii) those with positive results for influenza A, influenza B, RSV, or SARS-CoV-2 in any Tampere University Hospital Department regardless of test method, and (iii) those filling the first criterion and analyzed with a follow-up broad-panel respiratory test (respiratory viral, bacterial, or microbial panel) within 1 week after initial PoC testing. A more detailed description of the registry data collection process and available routine respiratory molecular diagnostic test methods is presented in supplementary data (Data S1 and S2). The collected data included the requested test and test result, patient age at the time of testing, and the hospital department requesting the test.

### Analysis

Defining the index test’s diagnostic (sensitivity, specificity, and accuracy) and analytical performance (sensitivity, specificity) against the reference tests was the primary endpoint of the study. As a secondary endpoint, the difference in the diagnostic yield of the index test and the comparator SARS-CoV-2- and RSV-triplex tests was assessed based on the paired result data of the original (triplex) and evaluated (tetraplex) tests. As another secondary endpoint, respiratory diagnostic practices of the Tampere University Hospital (registry data) were described to assess the relevance of the index test panel coverage in the clinical context of the study. Descriptive statistics (sensitivity, specificity, accuracy) were calculated using Microsoft Excel. Ninety-five percent confidence intervals were calculated using GraphPad QuickCalcs (GraphPad Software, Boston, MA, the United States).

## RESULTS

### Study participants

**Fig 1:**
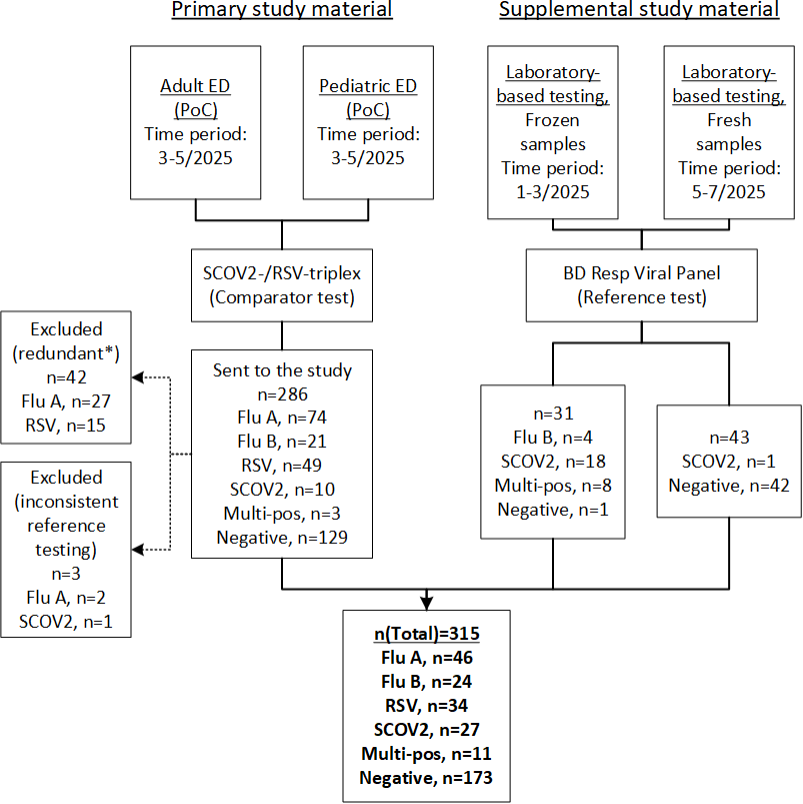
Case enrollment for diagnostic accuracy study. Samples sent to the evaluation laboratory by AED/PED were sorted according to the original result. After discontinuing AED/PED sample collection, samples positive for Flu A and RSV were excessive. Sample collection for other result classes was subsequently continued with laboratory-based testing samples (stored positive samples or fresh presumptive negative samples). Frequencies refer to testing results with the index test. *Exceeding the target sample number for each analyte. ED, emergency department; Flu A, influenza A; Flu B, influenza B; PoC, point-of-care; RSV, respiratory syncytial virus; SCOV2, SARS-CoV-2.

A total of 2,050 PoC samples were analyzed during the sample collection period (1,641 in AED, 409 in PED), 286 of which were sent to the evaluation laboratory. A total of 318 samples were analyzed, including 244 PoC samples and 74 laboratory-based samples (Fig. 1). Three PoC samples were excluded from the result analysis due to inconsistent reference testing (missing secondary Xpert analysis). Summary of patient demographics is presented in Table 1. Most samples (76.5%) were associated with PoC testing of the two emergency departments of Pirha, the rest being central laboratory testing samples. Most of the participants were pediatric patients (51.1%), followed by elderly patients (34.9%). Distribution of sex was roughly equal, with slightly more female participants (52.4%). Approximately half of the included samples were positive for at least one of the target pathogens with the reference test (47.3%).

**TABLE 1:** Demographic data of the study^*a*^

(*n*, total = 315)	*n*	%
Original test		
RSV-triplex only	20	6.3
SCOV2-triplex only	102	32.4
Both triplex tests	119	37.8
Laboratory test	74	23.5
Department		
Pediatric ED	161	51.1
Adult ED	82	26.0
Other	72	22.9
Sex		
Male	150	47.6
Female	165	52.4
Age		
0–5	118	37.5
6–15	43	13.7
16–64	44	14.0
65+	110	34.9
Reference test result		
Flu A	45	14.3
Flu B	20	6.3
RSV	42	13.3
SCOV2	27	8.6
Multi-positive	15	4.8
Negative	166	52.7

^*a*^ ED, emergency department; Flu A, influenza A; Flu B, influenza B; RSV, respiratory syncytial virus; SCOV2, SARS-CoV-2.

### Diagnostic accuracy

A total of 315 samples were included in the diagnostic accuracy analysis. Diagnostic accuracy results for the index test and the primary reference BD MAX test are summarized in Table 2. The index test detected every positive case present in the study, regardless of the target pathogen, whereas the BD MAX assay had sensitivities below 95% for detection of RSV and SARS-CoV-2. The specificity of the index test was below 100% for detection of any of the target pathogens. The lowest index test specificity—but reciprocally the lowest sensitivity of the BD MAX assay—was associated with detection of RSV. Discrepant results (*n* = 23) are described in the supplementary material (Supplemental material S3). The overall accuracy for detection of any of the four target pathogens was 98.6% (95% CI: 97.7%–99.1%) for the index test, and 99.4% (95% CI: 98.7%–99.7%) for the primary reference test, respectively. A simulation of the impact of different positivity rates on testing outcomes with the observed diagnostic accuracies is presented in Table 3.

**TABLE 2:** Diagnostic accuracy results^*a*^

Test	Target	TP	FN	TN	FP	Sensitivity, % (95% CI)	Specificity, % (95% CI)	Accuracy, % (95% CI)
Cobas Liat	Flu A	53	0	258	4	100 (91.9–100)	98.5 (96.0–99.6)	98.7 (96.7–99.6)
	Flu B	24	0	290	1	100 (83.7–100)	99.7 (97.9–100)	99.7 (98.0–100)
	RSV	50	0	257	8	100 (91.5–100)	97.0 (94.1–98.6)	97.5 (95.0–98.8)
	SCOV2	37	0	273	5	100 (88.8–100)	98.2 (95.7–99.4)	98.4 (96.2–99.4)
	Total	164	0	1078	18	100 (97.3–100)	98.4 (97.4–99.0)	98.6 (97.7–99.1)
BD MAX	Flu A	53	0	262	0	100 (91.9–100)	100 (98.3–100)	100 (98.6–100)
	Flu B	24	0	291	0	100 (83.7–100)	100 (98.4–100)	100 (98.6–100)
	RSV	46	4	265	0	92.0 (80.7–97.4)	100 (98.3–100)	98.7 (96.7–99.6)
	SCOV2	35	2	276	2	94.6 (81.4–99.4)	99.3 (97.2–99.9)	98.7 (96.7–99.6)
	Total	158	6	1094	2	96.3 (92.1–99.0)	99.8 (99.3–100)	99.4 (98.7–99.7)

^*a*^ CI, confidence interval; Flu A, influenza A; Flu B, influenza B; FN, false negative; FP, false positive; RSV, respiratory syncytial virus; SCOV2, SARS-CoV-2; TN, true negative; TP, true positive.

**TABLE 3:** Simulation of the impact of positivity rate on positive and negative predictive values with a hypothetical cohort (*n* = 500)^*a*^

Target	Accuracy	1% positivity rate	10% positivity rate	50% positivity rate
Flu A	Se: 100%	TP: 5/FN: 0	TP: 50/FN: 0	TP: 250/FN: 0
	Sp: 98.5%	TN: 487/FP: 8	TN: 443/FP: 7	TN: 246/FP: 4
		PPV: 38.5%/NPV: 100%	PPV: 87.7%/NPV: 100%	PPV: 98.4%/NPV: 100%
Flu B	Se: 100%	TP: 5/FN: 0	TP: 50/FN: 0	TP: 250/FN: 0
	Sp: 99.7%	TN: 493/FP: 2	TN: 448/FP: 2	TN: 249/FP: 1
		PPV: 71.4%/NPV: 100%	PPV: 96.2%/NPV: 100%	PPV: 99.6%/NPV: 100%
RSV	Se: 100%	TP: 5/FN: 0	TP: 50/FN: 0	TP: 250/FN: 0
	Sp: 97.0%	TN: 480/FP: 15	TN: 436/FP: 14	TN: 242/FP: 8
		PPV: 25%/NPV: 100%	PPV: 78.1%/NPV: 100%	PPV: 96.9%/NPV: 100%
SCOV2	Se: 100%	TP: 5/FN: 0	TP: 50/FN: 0	TP: 250/FN: 0
	Sp: 98.2%	TN: 486/FP: 9	TN: 442/FP: 8	TN: 246/FP: 4
		PPV: 35.7%/NPV: 100%	PPV: 86.2%/NPV: 100%	PPV: 98.4%/NPV: 100%

^*a*^ Flu A, influenza A; Flu B, influenza B; FN, false negative; FP, false positive; NPV, negative predictive value; PPV, positive predictive value; RSV, respiratory syncytial virus; SCOV2, SARS-CoV-2; Se, sensitivity; Sp, specificity; TN, true negative; TP, true positive.

**Fig 2:**
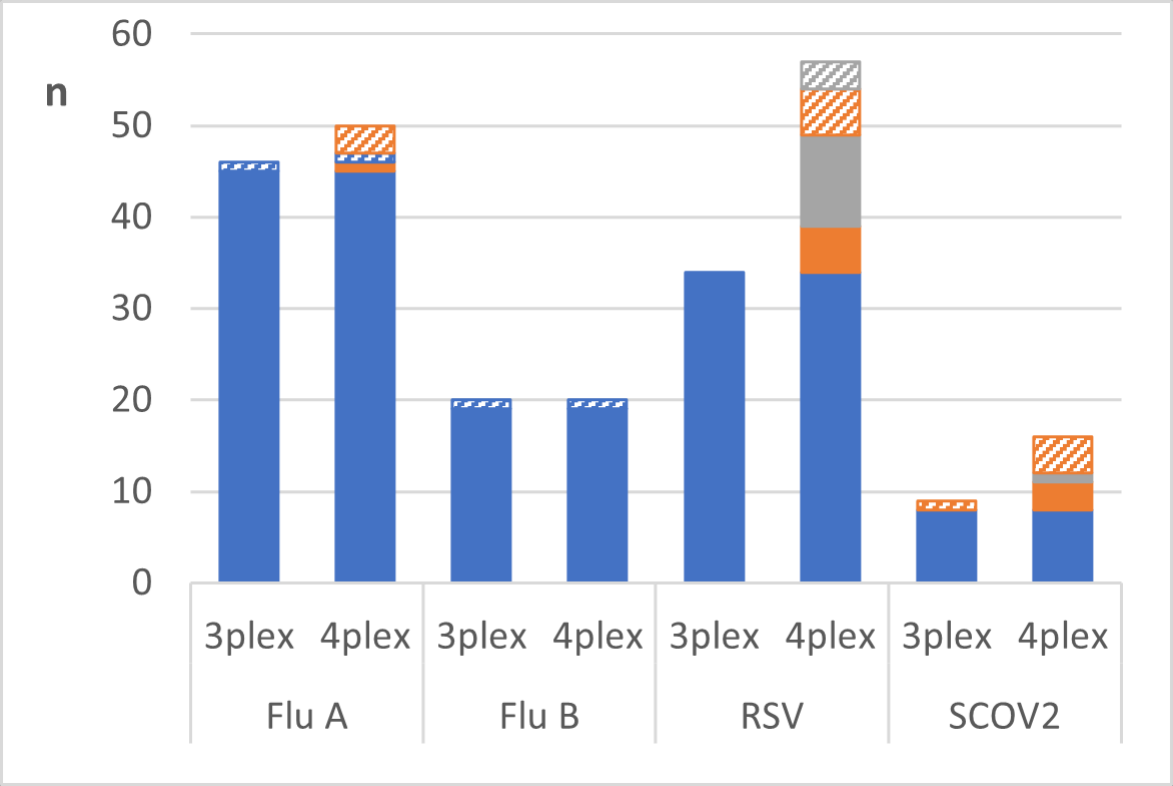
Diagnostic yield of the comparator triplex tests and the index tetraplex test. Results are based on original point-of-care triplex testing in emergency departments and subsequent testing with the index test. Blue bars indicate cases positive with both triplex and tetraplex testing, orange bars indicate cases positive only with triplex or tetraplex testing, and grey bars indicate positive cases not covered by the original triplex testing. Hatched bars (blue, orange, grey) indicate positive cases not confirmed by the reference tests. Flu A, influenza A; Flu B, influenza B; RSV, respiratory syncytial virus; SCOV2, SARS-CoV-2.

Based on comparison with the original triplex test results, the diagnostic yield of the index test was non-inferior to the comparator tests. Higher diagnostic yield with the index test was seen for influenza A, RSV, and SARS-CoV-2 (Fig. 2). A relatively high proportion of additional RSV-positive results (23% of all RSV-positive, *n* = 13) were associated with cases where RSV was not covered by the original triplex testing. Of the total added diagnostic yield, 57% (*n* = 20) was positive with reference testing. Two samples were positive with comparator and index tests but negative with reference testing.

### Analytical performance

Relative analytical sensitivity between the index and reference tests was assessed by analyzing a fivefold dilution series of a commercial tetraplex SARS-CoV-2, influenza A, influenza B, and RSV control sample (Table 4). In summary, Xpert was the least sensitive assay with positive results only for the initial dilution except for RSV, for which no positive result was observed. The Cobas Liat index test was the most sensitive assay for all analytes except for influenza B, for which the BD MAX assay was most sensitive.

**TABLE 4:** Relative analytical sensitivity results^*a*^^*,*^^*b*^

	SARS-CoV-2				Influenza A		
Dilution	Liat	Xpert	BD MAX	Dilution	Liat	Xpert	BD MAX
1:10	32.3	42.1	33.9	1:10	33.4	35.2; −	34.4
	34.9	37.8	35		34.9	32.2; 36.3	34
	34.2	39.5	33		34.4	34.1; 37	33.2
1:50	35.9	−	−	1:50	35.4	−	−
	38.7	−	−		35.6	−	−
	−	−	−		35.6	−	35.3
1:250	−	n/a	−	1:250	37.1	n/a	−
	39.7	n/a	−		37.4	n/a	−
	−	n/a	−		36.4	n/a	−
1:1250	−	n/a	−	1:1,250	−	n/a	−
	−	n/a	−		−	n/a	−
	−	n/a	−		−	n/a	−
							
	**Influenza B**				**RSV**		
**Dilution**	**Liat**	**Xpert**	**BD MAX**	**Dilution**	**Liat**	**Xpert**	**BD MAX**
1:10	32.4	38.1	29.1	1:10	33.1	−	37.8
	33.2	34	28.9		36.9	−	36.1
	32.7	35.0	29.1		35.7	−	−
1:50	34.8	−	34.4	1:50	−	−	−
	34	−	33.8		36.5	−	−
	35	−	33.8		36.6	−	−
1:250	−	n/a	38	1:250	−	n/a	−
	−	n/a	37.1		37.4	n/a	−
	−	n/a	37.3		35.9	n/a	−
1:1250	−	n/a	−	1:1,250	−	n/a	−
	−	n/a	−		−	n/a	−
	−	n/a	−		−	n/a	−

^*a*^ −, negative; n/a, not applicable (not analyzed); RSV, respiratory syncytial virus.

^*b*^ Positive results are presented as PCR cycle thresholds (Ct) to describe intra-assay characteristics.

Analytical specificity was evaluated using various clinical isolates and a commercial control panel. A total of 9 in-panel and 46 off-panel samples were tested, the off-panel samples, including 21 viral strains, 23 bacterial strains, and two fungal strains (S4). The index test showed expected positive results for all in-panel microbes and negative results for all off-panel microbes.

### Clinical context of the study (registry data analysis)

Testing practices of the emergency departments and respiratory diagnostic results of all Tampere University Hospital departments were examined to assess the validity of the index test’s analytical panel coverage.

A total of 10,032 valid, individual PoC respiratory tests were performed in AED and PED of Tampere University Hospital between July 2024 and June 2025, corresponding to 8,274 nasopharyngeal swab samples (Table 5). A total of 75.3% (*n* = 6 229) of all PoC testing was performed in AED, 24.7% (*n* = 2,045) in PED. Most of the AED testing involved SARS-CoV-2-triplex testing only (98.1%, *n* = 6,110), whereas most of the PED testing involved using both triplex tests (81.7%, *n* = 1,670). Regarding non-overlapping microbes, SARS-CoV-2 analysis was covered in 99.5% (*n* = 6,198) of AED testing and in 90.0% (*n* = 1,841) of PED testing, while RSV was covered in 1.91% (*n* = 119) of AED testing and 91.6% (*n* = 1,874) of PED testing, respectively.

**TABLE 5:** Performed PoC testing with respect to age group and emergency department at the study site between July 2024 and June 2025^*a*^

	Test	0−5 yo, *n* (%)	6−15 yo, *n* (%)	16−64 yo, *n* (%)	65+ yo, *n* (%)	Total, *n* (%)
Adult ED	RSV-triplex only			12 (0.2)	19 (0.3)	31 (0.5)
	SCOV2-triplex only	1 (<0.1)	1 (<0.1)	2,198 (35.3)	3,910 (62.8)	6,110 (97.6)
	Both			34 (0.5)	54 (0.9)	88 (1.4)
Pediatric ED	RSV-triplex only	162 (7.9)	42 (2.1)			204 (10.0)
	SCOV2-triplex only	62 (3.0)	108 (5.3)	1 (<0.1)		171 (8.4)
	Both	1,340 (65.5)	329 (16.1)	1 (<0.1)		1,670 (81.7)

^*a*^ Percentages correspond to ratios within an ED. ED, emergency department; RSV, respiratory syncytial virus; SCOV2, SARS-CoV-2; yo, years old.

**Fig 3:**
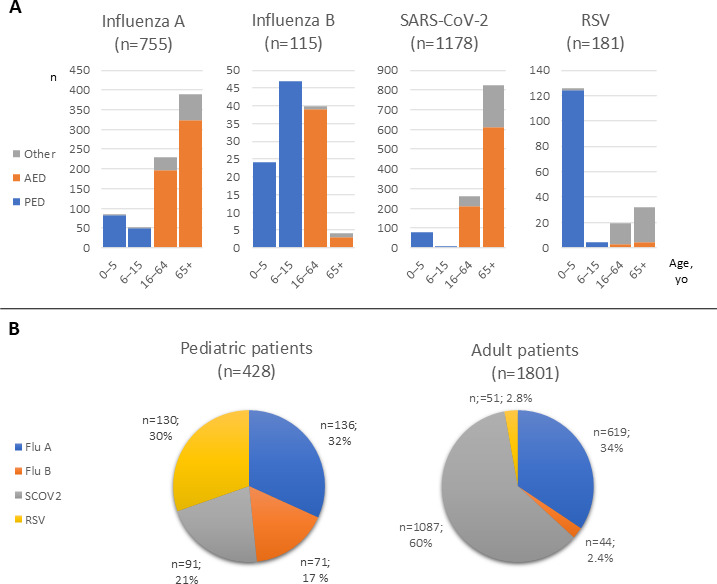
Distribution of influenza A, influenza B, SARS-CoV-2, and RSV findings in the registry data. (**A**) Bar charts show frequencies of positive findings. (**B**) Pie charts show proportions of positive findings (irrespective of the requesting hospital department). AED, adult emergency department; Flu A, influenza A; Flu B, influenza B; PED, pediatric emergency department; RSV, respiratory syncytial virus; SCOV2, SARS-CoV-2; yo, years old.

In terms of testing results, a total of 755 positive influenza A, 115 influenza B, 1,178 SARS-CoV-2, and 181 RSV results were recorded in any Tampere University Hospital department, irrespective of testing method (Fig. 3). Among pediatric patients, 96%–100% of all positive results for the four viruses were detected in PED, while among adult patients, influenza B was the only analyte that was almost completely detected in AED (Fig. 3). Most infrequently detected by ED testing, only 5.9% (*n* = 3/51) of positive RSV results were associated with PoC testing in AED.

**Fig 4:**
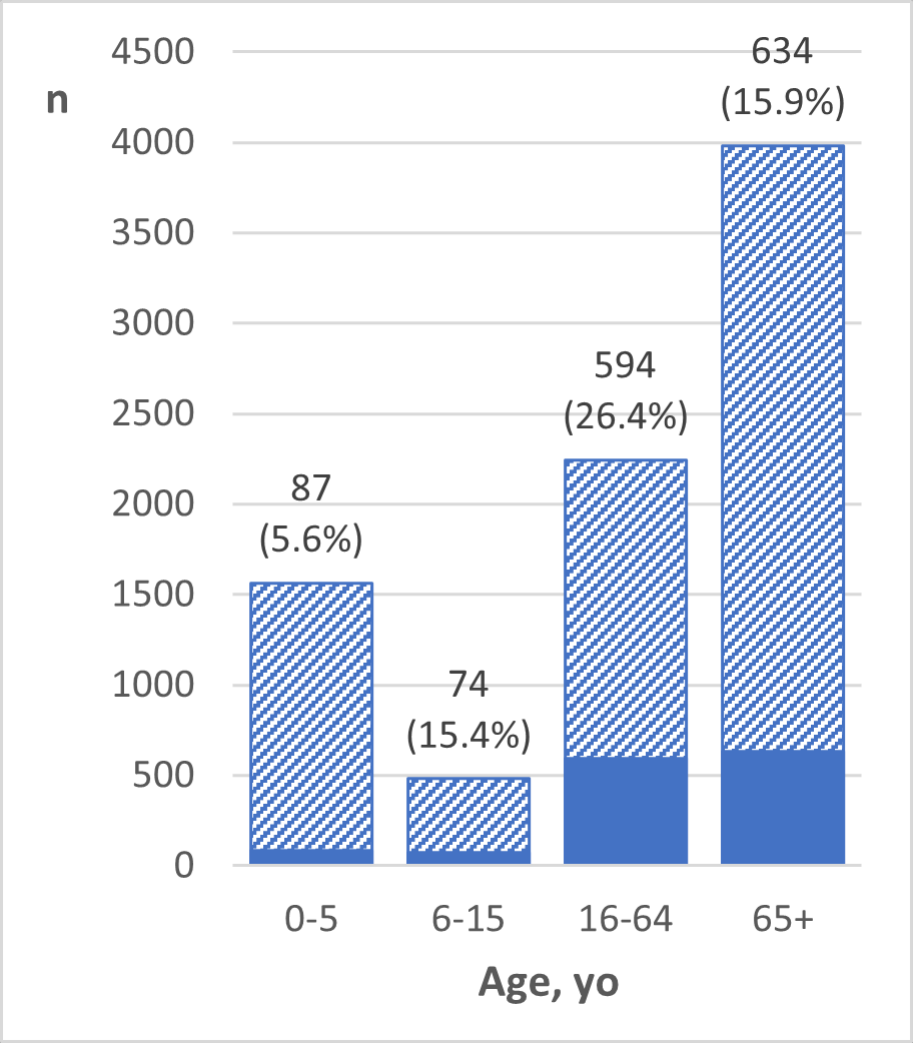
Follow-up testing practices after initial PoC testing. Overall, the height of bars represents the total number of performed PoC tests among the different age groups; the solid portion indicates the number of individuals receiving follow-up testing with broad-panel tests (with number and percentage displayed above the bar), while the hatched portion indicates the number of individuals who did not receive follow-up testing. yo, years old.

Follow-up testing with one or several broad-panel tests after initial PoC testing was applied in 16.8% (*n* = 1,389) of all PoC-tested cases. Follow-up testing was least frequent among young children (5.6%, *n* = 87), while working-aged patients were tested most frequently (26.4%, *n* = 594) (Fig. 4). Of the three available broad-panel tests, namely respiratory viral, respiratory bacterial, and respiratory microbial panels (S2), testing with the broadest test panel was most common among young children (46%, *n* = 49), with the rate decreasing sequentially with older age groups (S5). For all age groups except young children, respiratory bacterial panel was the most common broad-panel follow-up test (45%–51%). Follow-up testing with more than one test panel was common: an average of 1.6 different test panels were used per patient. The positivity rate of follow-up testing was highest among young children (71.3%) and decreased sequentially among older age groups (52.7%, 52.2%, and 30.8%, respectively). Follow-up testing yielded a total of 718 microbial findings. *Mycoplasma pneumoniae*, *Haemophilus influenzae*, and Human Rhinovirus were the three most common findings, respectively, accounting for over half of all microbial findings (Table 6). The most common finding among the index test in-panel microbes was RSV (*n* = 34, 4.7%), followed by SARS-CoV-2 (*n* = 18, 2.5%), influenza A (*n* = 14, 1.9%), and influenza B (*n* = 2, 0.3%), respectively.

**TABLE 6:** Microbial findings of follow-up testing with broad-coverage test panels after initial PoC testing

Microbe (*n* = 718)	*n* (%)
*Mycoplasma pneumoniae*	200 (27.9)
*Haemophilus influenzae*	126 (17.5)
Human Rhinovirus	85 (11.8)
*Streptococcus pneumoniae*	81 (11.3)
Metapneumovirus	53 (7.4)
Rhinovirus/Enterovirus	34 (4.7)
Respiratory syncytial virus	34 (4.7)
Adenovirus	28 (3.9)
SARS-CoV-2	18 (2.5)
Influenza A virus	14 (1.9)
Parainfluenzavirus	12 (1.7)
Bocavirus	9 (1.3)
Enterovirus	9 (1.3)
Human coronavirus OC43	8 (1.1)
*Bordetella pertussis*	2 (0.3)
Influenza B virus	2 (0.3)
Human coronavirus NL63	2 (0.3)
*Chlamydia pneumoniae*	1 (0.1)

## DISCUSSION

In this study, we evaluated the diagnostic accuracy of the Cobas Liat SARS-CoV-2, influenza A/B, and RSV assay and its applicability in adult and pediatric emergency department patients. Together with the Cobas Liat system, they form a simple, fast, and compact PoC-compatible test method for the detection of the four most important epidemic respiratory viruses. In addition to combining the coverage of the two predecessor Cobas respiratory triplex tests, the new assay also employs Uracil-DNA glycosylase to reduce the risk of amplicon carryover contamination. Assay design is otherwise similar to the earlier triplex tests, including an internal process control instead of an increasingly employed endogenous internal control.

Although initial diagnostic accuracy evaluation suggested reduced specificity compared to the primary reference test, BD MAX Respiratory Viral Panel, further verification analyses with the Xpert Xpress CoV-2/Flu/RSV plus assay, together with analytical sensitivity analyses, in fact, suggest that the index test was the most sensitive assay of the study. This is a somewhat surprising result as the accessibility and convenience of PoC testing have been traditionally thought to come with a trade-off in sensitivity compared to more complex laboratory-based assays used in centralized testing, although the difference has decreased in the era of molecular PoC testing (13, 14). Although these results suggest potential as a rule-out test, this could not be assessed due to the unavailability of comprehensive clinical data.

At the time of the study, PoC testing for respiratory infections at the two emergency departments involved the use of two partially overlapping predecessor triplex assays. The use of one tetraplex test instead may provide two main benefits. First, the use of one test offers a streamlined workflow, decreasing reagent costs and hands-on time compared to systematic double testing. In the context of this study, the testing practices of PED would substantially benefit from the implementation of a tetraplex test, as most PED testing events involve double testing. Specifically, double testing either increases total turnaround time from about 20 to 40 min or halves analytical capacity, which is usually minimal for the single-module Cobas Liat system. To a minor extent, use of a single test may also prevent confusion related to inconsistent results of overlapping analytes and the risk of performing the wrong test. Specific assay prices are essentially dependent on testing volumes. In our setting, the cost of a single tetraplex test was approximately 1.5 times that of a single triplex test, or 0.75 times that of two separate triplex tests. This implies that while implementing tetraplex testing in PED would lead to considerable savings in reagent costs, the additional RSV result would not come free for AED. Based on observed testing rates, estimated savings at PED and additional costs at AED could be around 15,000 and 60,000 € per year, respectively (data not shown).

Second, the use of a tetraplex test may reveal diagnostic gaps if double testing is not systematically performed. Especially, RSV testing in adult patients is often overlooked. Although RSV causes significant morbidity and mortality in the elderly and the immunocompromised, it is often underdiagnosed due to low clinical suspicion, lack of routine testing, and lack of specific treatment (15). Indeed, almost 95% of all adult RSV cases in the registry analysis were associated with other than AED PoC testing, and nearly 30% of all hospitalized RSV cases in the study were among adults. These results suggest that implementation of the index tetraplex test would substantially increase the number of detected RSV cases in the AED. Altogether, the number of all adult RSV cases in the Tampere University Hospital corresponded to 0.8% of all PoC testing in AED. Although the importance of RSV in the elderly is increasingly recognized (16), more data are needed to show the relationship between the detection of RSV and improved treatment outcomes. Otherwise, the additional RSV result may simply represent an added cost without a clear benefit in AED PoC testing. Detection of respiratory viruses has been reported to decrease unnecessary prescription of antibiotics, but depending on local antimicrobial stewardship practices, such benefit may not always be seen (17). As RSV is the only one of the index test’s target viruses that is not associated with specific antiviral treatment, the most evident benefit of emergency department RSV testing would be related to cohorting of patients admitted to the ward. In this sense, local cohorting practices should be scrutinized to assess the relevance of tetraplex testing. Essentially, if admitted patients are not cohorted or are always placed in single-patient rooms, specific RSV infection status may provide limited benefit. In other cases, the SARS-CoV-2-triplex single-test approach may pose a significant risk for transmitting and contracting RSV among individuals who tested negative for influenza A, B, or SARS-CoV-2. Notably, limiting tetraplex testing to high-risk patients to reduce additional costs is not recommended for patient cohorting unless these patients are isolated from patients tested only with the SARS-CoV-2-triplex test. However, as noted earlier, this diagnostic approach would complicate choosing the right PoC test.

In addition to clinical aspects, gaining epidemiological data would be an added benefit of systematic tetraplex testing. This study provides estimates of adult RSV infection rates based only on sporadic RSV testing. Systematic data on RSV infections would facilitate the evaluation of the burden of RSV infections in ED patients. Gradually, such data will inevitably increase as commercial tetraplex tests (or higher-level multiplex tests) are becoming increasingly common over triplex tests.

To assess the impact of broader panel analysis more elaborately, follow-up testing practices of PoC-tested patients were examined. Such testing typically covers other clinically relevant respiratory viruses and sometimes bacteria (S2). Unexpectedly, follow-up testing was most common among working-aged patients, and least common among young children. Follow-up testing practices, especially among young children, suggest that, in general, systematic PoC testing with a tetraplex panel, such as the index test, may be sufficient instead of a more expensive broad-panel test. Distinct testing practices among older age groups cannot be explained simply by the lack of RSV in initial PoC testing. Notably, the analysis period of the registry study encompassed exceptional epidemic peaks for adenovirus, *Bordetella pertussis*, and *Mycoplasma pneumoniae*. Based on positive result data and especially for *M. pneumoniae*, these epidemics distorted typical seasonal epidemiology and likely influenced routine practices for respiratory infection diagnostics, especially toward more frequent follow-up testing with broad-panel tests. However, this phenomenon is inherent to the field—with a totally different threshold, SARS-CoV-2 was responsible for a similar phenomenon a few years earlier. Excluding the effect of epidemics may not be possible in a rapidly evolving diagnostic field, such as respiratory molecular diagnostics. Of note, the role of laboratory-based testing in such cases should be highlighted, being more versatile in terms of capacity and test repertoire than PoC testing.

The Cobas Liat system is a CLIA-waived *in vitro* diagnostic application, especially for PoC use. For this reason, the system (workflow, instrument, user interface) has an oversimplified design. The user interface version employed in this study did not display cycle threshold values or amplification curves. In the context of PoC use by nurses or other non-laboratory-based healthcare staff, this data may not be relevant. However, local examples have shown that such information may be valuable in troubleshooting, e.g., for exposing systematic errors due to instrument malfunctions (data not shown). In the setting of this study, PoC user departments are supervised by the local central laboratory with specialists for clinical microbiology and molecular diagnostics. In the context of this study, and with the Roche technical support available, no such problematic cases were observed.

This study had several limitations. Due to the realized study period, frozen samples had to be used. This may have had deteriorating effects on the analyzed samples. However, except for the Cobas triplex assays, index and reference test analyses were performed at the same thawing cycle for all samples. Therefore, the setting was equal and not biased toward the index test or any reference tests. By contrast, the sensitivities of the index test compared with the comparator triplex tests may be underestimated.

As a convenience sample was collected, the authentic incidence was not reproduced in the study material. Positive (PPV) or negative predictive values (NPV) could therefore not be calculated. However, a simulation with different epidemiological situations was provided. The specificity rates based on comparison with reference tests show that while the estimated PPVs and NPVs are high during the epidemic season, off-season PPV rates are severely impaired. However, as the analytical sensitivity of the index test was higher than that of any of the reference tests for SARS-CoV-2, influenza A, and RSV, some cases classified as false positives by the index test may, in fact, represent true positives that were not detected by the reference tests due to their higher limits of detection. Hence, the presented comparison likely underestimates the index test’s PPV rates outside the epidemic season. Altogether, the results suggest high applicability as a rule-in test during the epidemic season. The assay may also have potential as a rule-in test outside the epidemic season, but this could not be verified in this study due to the inferior analytical sensitivity of the reference tests.

Although samples were collected in two emergency departments, this study was essentially a monocenter study. Although associated viral strains were not identified, a multicenter study would likely have captured a more comprehensive representation of different virus strains. The circulating virus types during the epidemic season 2024/2025 in Finland are described in the supplementary material (Supplemental material S6).

Clinical data were not available in this study except for the initial inclusion criterion of respiratory infection symptoms. Therefore, the index test’s potential as a rule-out test could not be reliably assessed. However, as there were no positive cases missed by the index test, follow-up testing with a laboratory-based test would likely be redundant. The lack of clinical data may also have had some effect on the collected data. For example, some of the pediatric patients tested only with the SARS-CoV-2-triplex test in PED may have only recently been tested for RSV elsewhere. Similarly, the broad-panel follow-up testing analysis may be an overestimation, as the possibility of new infections after initial PoC testing cannot be excluded.

### Conclusions

The evaluated Cobas Liat SARS-CoV-2, influenza A/B, and RSV assay demonstrated higher diagnostic sensitivity but lower specificity compared to the primary reference test, the BD MAX Respiratory Viral Panel assay. However, lower specificity is likely explained by superior analytical sensitivity compared to any reference tests. The assay is suitable as a rule-in test for respiratory infections by all four viruses. Although suitability as a rule-out test could not be reliably assessed in this study, our results suggest that due to excellent sensitivity, it is unnecessary to control negative results with corresponding laboratory-based tests. A diagnostic process involving systematic double testing with Cobas SARS-CoV-2- and RSV-triplex tests should be replaced by single testing with the index test. In settings with other triplex-testing algorithms, cost-efficiency is more dependent on the ratio of double testing, local RSV/SARS-CoV-2 epidemiology, and customer-specific reagent costs. Based on local follow-up testing practices, the tetraplex panel seems to be sufficient for systematic testing in emergency departments instead of broad-panel tests.

## SUPPLEMENTAL MATERIAL

Supplemental materials jcm.00274-26-s0001.docx Supplemental materials.

ASM does not own the copyrights to Supplemental Material that may be linked to, or accessed through, an article. The authors have granted ASM a non-exclusive, world-wide license to publish the Supplemental Material files. Please contact the corresponding author directly for reuse.

## References

[1] Kyu HH, Vongpradith A, Sirota SB, Novotney A, Troeger CE, Doxey MC, Bender RG, Ledesma JR, Biehl MH, Albertson SB, et al.. 2022. Age–sex differences in the global burden of lower respiratory infections and risk factors, 1990–2019: results from the global burden of disease study 2019. Lancet Infect Dis 22:1626–1647. doi:10.1016/S1473-3099(22)00510-2 35964613 PMC9605880

[2] Schanzer DL, Saboui M, Lee L, Nwosu A, Bancej C. 2018. Burden of influenza, respiratory syncytial virus, and other respiratory viruses and the completeness of respiratory viral identification among respiratory inpatients, Canada, 2003‐2014. Influenza Resp Viruses 12:113–121. doi:10.1111/irv.12497 PMC5818333 29243369

[3] Novel Swine-Origin Influenza A (H1N1) Virus Investigation Team. 2009. Emergence of a novel swine-origin influenza A (H1N1) virus in humans. N Engl J Med 360:2605–2615. doi:10.1056/NEJMoa0903810 19423869

[4] Huang C, Wang Y, Li X, Ren L, Zhao J, Hu Y, Zhang L, Fan G, Xu J, Gu X, et al.. 2020. Clinical features of patients infected with 2019 novel coronavirus in Wuhan, China. The Lancet 395:497–506. doi:10.1016/S0140-6736(20)30183-5 PMC7159299 31986264

[5] Manirambona E, Okesanya OJ, Olaleke NO, Oso TA, Lucero-Prisno DE. 2024. Evolution and implications of SARS-CoV-2 variants in the post-pandemic era. Discov Public Health 21:16. doi:10.1186/s12982-024-00140-x

[6] Nair H, Nokes DJ, Gessner BD, Dherani M, Madhi SA, Singleton RJ, O’Brien KL, Roca A, Wright PF, Bruce N, Chandran A, Theodoratou E, Sutanto A, Sedyaningsih ER, Ngama M, Munywoki PK, Kartasasmita C, Simões EAF, Rudan I, Weber MW, Campbell H. 2010. Global burden of acute lower respiratory infections due to respiratory syncytial virus in young children: a systematic review and meta-analysis. Lancet 375:1545–1555. doi:10.1016/S0140-6736(10)60206-1 20399493 PMC2864404

[7] Zheng Z, Warren JL, Shapiro ED, Pitzer VE, Weinberger DM. 2022. Estimated incidence of respiratory hospitalizations attributable to RSV infections across age and socioeconomic groups. Pneumonia (Nathan) 14:6. doi:10.1186/s41479-022-00098-x 36280891 PMC9592130

[8] Kaushik N, Khangulov VS, O’Hara M, Arnaout R. 2018. Reduction in laboratory turnaround time decreases emergency room length of stay. Open Access Emerg Med 10:37–45. doi:10.2147/OAEM.S155988 29719423 PMC5916382

[9] Vrijsen BEL, Haitjema S, Westerink J, Hulsbergen-Veelken CAR, van Solinge WW, Ten Berg MJ. 2022. Shorter laboratory turnaround time is associated with shorter emergency department length of stay: a retrospective cohort study. BMC Emerg Med 22:207. doi:10.1186/s12873-022-00763-w 36544114 PMC9768765

[10] Yin N, Van Nuffelen M, Bartiaux M, Préseau T, Roggen I, Delaunoy S, Mahadeb B, Dahma H, Busson L, Vandenberg O, Hallin M. 2022. Clinical impact of the rapid molecular detection of RSV and influenza A and B viruses in the emergency department. PLoS One 17:e0274222. doi:10.1371/journal.pone.0274222 36054246 PMC9439204

[11] Au Yeung V, Thapa K, Rawlinson W, Georgiou A, Post JJ, Overton K. 2021. Differences in antibiotic and antiviral use in people with confirmed influenza: a retrospective comparison of rapid influenza PCR and multiplex respiratory virus PCR tests. BMC Infect Dis 21:321. doi:10.1186/s12879-021-06030-w 33827458 PMC8024678

[12] Alsharksi AN, Sirekbasan S, Gürkök-Tan T, Mustapha A. 2024. From tradition to innovation: diverse molecular techniques in the fight against infectious diseases. Diagnostics (Basel) 14:2876. doi:10.3390/diagnostics14242876 39767237 PMC11674978

[13] Gentilotti E, De Nardo P, Cremonini E, Górska A, Mazzaferri F, Canziani LM, Hellou MM, Olchowski Y, Poran I, Leeflang M, Villacian J, Goossens H, Paul M, Tacconelli E. 2022. Diagnostic accuracy of point-of-care tests in acute community-acquired lower respiratory tract infections. A systematic review and meta-analysis. Clin Microbiol Infect 28:13–22. doi:10.1016/j.cmi.2021.09.025 34601148

[14] Zu Y, Chang H, Cui Z. 2025. Molecular point-of-care testing technologies: current status and challenges. Nexus 2:100059. doi:10.1016/j.ynexs.2025.100059

[15] Branche AR. 2019. Why making a diagnosis of respiratory syncytial virus should matter to clinicians. Clin Infect Dis 69:204–206. doi:10.1093/cid/ciy880 30321317

[16] Begley KM, Monto AS, Lamerato LE, Malani AN, Lauring AS, Talbot HK, Gaglani M, McNeal T, Silveira FP, Zimmerman RK, Middleton DB, Ghamande S, Murthy K, Kim L, Ferdinands JM, Patel MM, Martin ET. 2023. Prevalence and clinical outcomes of respiratory syncytial virus vs influenza in adults hospitalized with acute respiratory illness from a prospective multicenter study. Clin Infect Dis 76:1980–1988. doi:10.1093/cid/ciad031 36694363 PMC10250013

[17] Saarela E, Tapiainen T, Kauppila J, Pokka T, Uhari M, Kauma H, Renko M. 2020. Impact of multiplex respiratory virus testing on antimicrobial consumption in adults in acute care: a randomized clinical trial. Clin Microbiol Infect 26:506–511. doi:10.1016/j.cmi.2019.09.013 31574339 PMC7128925

