# Supplementary material for: From triplex to tetraplex: evaluation of the diagnostic accuracy of the new Roche Cobas Liat SARS-CoV-2, influenza A/B & RSV assay
Source: J Clin Microbiol. 2026 Jul 20;64(8):e00274-26. doi: 10.1128/jcm.00274-26 (PMC13463847; doi:10.1128/jcm.00274-26)
Supplement: Supplementary file 1 [file jcm.00274-26-s0001.docx]

**S1. Description of processing and case enrollment of the registry data.**

Data 1. Number of PoC tests

Patient-level data was collected from the Fimlab data lake, with patient identifier pseudonymized. The inclusion period was July 2024 to June 2025. Only tests performed by the Adult (AED) or Pediatric Emergency Department (PED) of Tampere University Hospital were included. Only RSV-triplex or SARS-CoV-2-triplex tests were included. The number of RSV- and SARS-CoV-2-triplex testing events was retrieved, together with associated data on emergency department, age group (0–5, 6–15, 16–64, ≥65 years old) and test performed. A single testing event was defined as testing for RSV-triplex, SARS-CoV-2-triplex, or both tests on the same day. Only cases with valid results were included; invalid or cancelled tests were excluded. Multiple testing events per patient were allowed and counted separately.

Data 2. Number of influenza A, influenza B, RSV, and SARS-CoV-2 findings

Patient level data was collected from the Fimlab data lake, with patient identifier pseudonymized. The inclusion period was July 2024 to June 2025. Only tests performed by Tampere University Hospital departments were included. All tests containing influenza A, influenza B, RSV, and SARS-CoV-2 in the test panel were included (S2). Only cases with positive results for influenza A, influenza B, RSV, or SARS-CoV-2 were included. The number of positive cases for each pathogen was retrieved, together with associated data on pathogen, department (AED, PED, other), and age group (0–5, 6–15, 16–64, ≥65 years). Multiple different pathogen findings per patient were allowed and counted separately.

Data 3. Cases with broad-panel follow-up testing after initial PoC-testing.

Patient-level data was collected from the Fimlab data lake, with patient identifier pseudonymized. Only cases included in Data 1 were included. Cases with a broad-panel follow-up test (S2: R-Vir, R-Bact, or R-Mic) performed within seven days of the initial PoC test event were included. Only cases in which follow-up testing was performed (valid or invalid test results) were included; cancelled requests were excluded. If multiple follow-up tests were performed within the seven-day period, only the first test per panel was included. Each unique test identifier was allowed only once to prevent duplication due to adjacent PoC test events for the same patient. The numbers of follow-up tested cases (any panel), individual tests, and detected findings were retrieved, together with associated data on test panel, finding, and age group (0–5, 6–15, 16–64, ≥65 years).

**S2.** Target panels of molecular diagnostic assays used in the routine diagnostics of respiratory infection in the study setting.

| **Microbe** | **3plex-RP (SCOV2)** | **3plex-RP (RSV)** | **SCOV2** | **4plex-RP** | **R-Vir** | **R-Bact** | **R-Mic** |
| --- | --- | --- | --- | --- | --- | --- | --- |
| Influenza A | x | x |  | x | x^3^ |  | x^3^ |
| Influenza B | x | x |  | x | x |  | x |
| Severe Acute Respiratory Syndrome Coronavirus 2 (SARS-CoV-2) | x |  | x | x |  |  | x |
| Respiratory Syncytial Virus (RSV) |  | x |  | x | x^3^ |  | x |
| Adenovirus |  |  |  |  | x |  | x |
| Human Bocavirus |  |  |  |  | x |  | x |
| Coronavirus 229E |  |  |  |  | x |  | x^1^ |
| Coronavirus HKU1 |  |  |  |  |  |  | x^1^ |
| Coronavirus NL63 |  |  |  |  | x |  | x^1^ |
| Coronavirus OC43 |  |  |  |  | x |  | x^1^ |
| Enterovirus |  |  |  |  | x |  | x^2^ |
| Human Metapneumovirus |  |  |  |  | x |  | x |
| Human Rhinovirus |  |  |  |  | x |  | x^2^ |
| Middle East Respiratory Syndrome Coronavirus (MERS-CoV-2) |  |  |  |  |  |  | x |
| Parainfluenza Virus 1 |  |  |  |  | x |  | x^1^ |
| Parainfluenza Virus 2 |  |  |  |  | x |  | x^1^ |
| Parainfluenza Virus 3 |  |  |  |  | x |  | x^1^ |
| Parainfluenza Virus 4 |  |  |  |  | x |  | x^1^ |
| *Bordetella parapertussis* |  |  |  |  |  | x | x |
| *Bordetella pertussis* |  |  |  |  |  | x | x |
| *Chlamydia pneumoniae* |  |  |  |  |  | x | x |
| *Haemophilus influenzae* |  |  |  |  |  | x |  |
| *Legionella pneumoniae* |  |  |  |  |  | x | x |
| *Mycoplasma pneumoniae* |  |  |  |  |  | x | x |
| *Streptococcus pneumoniae* |  |  |  |  |  | x |  |

^1^ Reported individually by the assay but as a group (Coronavirus or Parainfluenza Virus) in the study registry data

^2^ Reported as a group (Human Rhinovirus/Enterovirus) by the assay and in the study registry data

^3^ Includes subtyping results by the test not reported in the study registry data

3plex-RP (SCOV2): Cobas® SARS-CoV-2 & Influenza A/B, Roche Molecular (point-of-care)

3plex RP (RSV): Cobas Influenza A/B & RSV assays, Roche Molecular (point-of-care)

SCOV2: Cobas SARS-CoV-2, Roche Molecular (central laboratory)

4plex-RP: BD Respiratory Viral Panel, BD or Xpert Xpress CoV-2/Flu/RSV plus, Cepheid (central laboratory)

R-Vir: Allplex Respiratory Panel 1-3, Seegene, Seoul, Korea (central laboratory)

R-Bact: Allplex Respiratory Panel 4, Seegene, Seoul, Korea (central laboratory)

R-Mic: QIAStat Respiratory SARS-CoV-2 Panel, QIAGEN, Hilden, Germany (central laboratory)

**S3.** Description of discordant results. Results discordant with the reference result are bolded.

| **n** | **Cobas 4plex (Ct)** | **BD MAX (Ct)** | **Xpert (Ct)** | **Reference result** | **Original Test** | **Original result** |
| --- | --- | --- | --- | --- | --- | --- |
| 1 | RSV (34.7)  SCOV2 (34.0) | **NEG** | RSV (39.8)  SCOV2 (43.0) | RSV  SCOV2 | RSV-triplex | NEG |
| 2 | **Flu A (35.2)** | NEG | NEG | NEG | Both | Flu A |
| 3 | Flu B (21.2)  RSV (31.4) | Flu B (22.6) **-** | Flu B (21.8)  RSV (38.0) | Flu B  RSV | Both | Flu B - |
| 4 | **Flu B (35.2)** | NEG | NEG | NEG | SCOV2-triplex | Flu B |
| 5 | RSV (12.2) - | RSV (15.4)  **SCOV2 (35.6)** | RSV (16.0) - | RSV - | Both | RSV - |
| 6 | **RSV (36.0)  SCOV2 (34.7)** | NEG | NEG | NEG | Both | NEG |
| 7 | **RSV (36.6)  SCOV2 (23.4)** | - SCOV2 (26.3) | **NEG** | - SCOV2 | Both | - SCOV2 |
| 8 | **RSV (36.4)** | NEG | NEG | NEG | SCOV2-triplex | NEG |
| 9 | RSV (30.8) | **NEG** | RSV (32.2) | RSV | Both | RSV |
| 10 | **RSV (36.2)** | NEG | NEG | NEG | Both | NEG |
| 11 | **RSV (34.3)** | NEG | NEG | NEG | SCOV2-triplex | NEG |
| 12 | **Flu A (36.6)**  Flu B (13.8)  RSV (29.8) | -  Flu B (15.4)  RSV (34.8) | -  Flu B (14.7)  RSV (37.6) | -  Flu B  RSV | Both | - Flu B - |
| 13 | **RSV (35.0)** | NEG | NEG | NEG | SCOV2-triplex | NEG |
| 14 | RSV (37.8)  **SCOV2 (12.5)** | RSV (15.5) - | RSV (17.3) - | RSV - | Both | RSV - |
| 15 | **SCOV2 (34.7)** | NEG | NEG | NEG | Both | NEG |
| 16 | RSV (17.0) - | RSV (19.9)  **SCOV2 (37.3)** | RSV (21.6) - | RSV - | RSV-triplex | RSV n/a |
| 17 | RSV (17.2)  SCOV2 (28.4) | RSV (39.6) **-** | RSV (20.2)  SCOV2 (35.9) | RSV  SCOV2 | Both | RSV  SCOV2 |
| 18 | **SCOV2 (35.2)** | NEG | NEG | NEG | Both | NEG |
| 19 | **Flu A (36.2)  RSV (35.6)** | NEG | NEG | NEG | RSV-triplex | NEG |
| 20 | RSV (35.7) | **NEG** | RSV (37.9) | RSV | SCOV2-triplex | NEG |
| 21 | **RSV (34.4)** | NEG | NEG | NEG | Both | NEG |
| 22 | **Flu A (35.9)** | NEG | NEG | NEG | Both | NEG |
| 23 | **SCOV2 (35.0)** | NEG | NEG | NEG | n/a | n/a |

-: Negative (for a given analyte); Ct: (PCR) cycle threshold; Flu A: influenza A; Flu B: influenza B; n/a: not applicable (not tested); NEG: negative (overall result); RSV: respiratory syncytial virus; SCOV2: SARS-CoV-2

**S4.** Analytical specificity panel microbes analyzed with the index test.

| **Microbe** | **Strain/subtype** | **Result** | **Source** |
| --- | --- | --- | --- |
| ***In-panel microbes*** |  |  |  |
| Influenza A | H1N1 (pdm09) | Flu A | 1 |
| Influenza A | H1N1 (New Caledonia/20/99) | Flu A | 1 |
| Influenza A | H3N2 | Flu A | 1 |
| Influenza B | Michigan/01/2021 | Flu B | 1 |
| RSV | Subtype A | RSV | 1 |
| RSV | Subtype A (A.D.1 NY Wad) | RSV | 1 |
| RSV | Subtype B | RSV | 1 |
| SARS-CoV-2 | B.1.1.529 (Omikron) | SCOV2 | 1 |
| SARS-CoV-2 | Omikron USA/MD-HP20874/2021 | SCOV2 | 1 |
| ***Off-panel microbes*** |  |  |  |
| Adenovirus | Type 1 | - | 1 |
| Adenovirus | Type 4 | - | 1 |
| Adenovirus | Type 14 | - | 1 |
| Bokavirus | Recombinant (Lambda) | - | 1 |
| Coronavirus | NL63 | - | 1 |
| Coronavirus | OC43 | - | 1 |
| Coronavirus | HKU1 | - | 1 |
| Cytomegalovirus | AD-169 | - | 2 |
| Echovirus | Type 11 | - | 2 |
| Enterovirus | D68 B3 | - | 1 |
| Herpes simplex virus 1 (HSV-1) | MacIntyre | - | 2 |
| Herpes simplex virus 2 (HSV-2) | MS | - | 2 |
| Human herpes virus 6 (HHV-6) | Z29 | - | 2 |
| Metapneumovirus | Type A1 | - | 1 |
| Metapneumovirus | Type B2 | - | 1 |
| Parainfluenza | 1 | - | 1 |
| Parainfluenza | 3 | - | 1 |
| Parechovirus | Type 1a | - | 2 |
| Rhinovirus | Type 3 | - | 1 |
| Rhinovirus | Type 5 | - | 1 |
| Varicella zoster virus (VZV) | Ellen | - | 2 |
| *Bordetella pertussis* | A639 | - | 1 |
| *Bordetella parapertussis* | A747 | - | 1 |
| *Chlamydia pneumoniae* |  | - | 1 |
| *Escherichia coli* | 0.1285; O18:H7:K1 | - | 2 |
| *Haemophilus influenzae* | MinnA | - | 2 |
| *Haemophilus parainfluenzae* |  | - | 3 |
| *Klebsiella oxytoca* |  | - | 3 |
| *Legionella pneumophila* | Serogroup 1 | - | 1 |
| *Listeria moncytogenes* | Serotype 1/2b | - | 2 |
| *Moraxella catarrhalis* |  | - | 3 |
| *Moraxella nonliquefaciens* |  | - | 3 |
| *Mycoplasma pneumoniae* |  | - | 1 |
| *Neisseria flava* |  | - | 3 |
| *Neisseria meningitidis* | Serogroup A | - | 2 |
| *Proteus mirabilis* |  | - | 3 |
| *Pseudomonas aeruginosa* |  | - | 3 |
| *Rothia mucilaginosa* |  | - | 3 |
| *Staphylococcus aureus* |  | - | 3 |
| *Staphylococcus lugdunensis* |  | - | 3 |
| *Streptococcus agalactiae* | Z019 | - | 2 |
| *Streptococcus dysgalactiae* |  | - | 3 |
| *Streptococcus pneumoniae* | Z022 | - | 2 |
| *Streptococcus viridans* |  | - | 3 |
| *Candida albicans* |  | - | 3 |
| *Cryptococcus gattii* | Z156 | - | 2 |

* Subtype/strain information as provided by the manufacturer/supplier.
1: External quality assessment sample
2: ZeptoMetrix NATMEC-BIO commercial control
3: Clinical isolate, identified using MALDI-TOF
-: negative; Flu A: influenza A; Flu B: influenza B; RSV: respiratory syncytial virus; SCOV2: SARS-CoV-2

**S5.** Use of broad-panel test panels in follow-up testing after initial PoC testing among different age groups.

yo: years old

**S6.** Description of circulating viruses in Finland in the epidemic season 2024-2025 (N. Ikonen, personal communication, January 16 2026).

**Influenza A**

A(H1N1)pdm09, predominant strain

Clade 5a.2a, predominant group C.1.9.3

Clade 5a.2a.1, predominant group D.3.1

A(H3N2)

Clade 2a.3a.1 (all cases), predominant groups J2 and J2.2

**Influenza B**

B/Victoria (all cases)

Clade V1A.3a.2 (all cases), groups C.5.1, C.5.6, C.5.6.1, and C.5.7

**RSV**

Data not available

**SARS-CoV-2**

Variant Omicron BA.2.86 (all cases)

Sublineage JN1.1
